# Osteosarcopenic Obesity: Current Knowledge, Revised Identification Criteria and Treatment Principles

**DOI:** 10.3390/nu11040747

**Published:** 2019-03-30

**Authors:** Owen J. Kelly, Jennifer C. Gilman, Dario Boschiero, Jasminka Z. Ilich

**Affiliations:** 1 Abbott Nutrition, Columbus, OH 43215, USA; jennifer.gilman@abbott.com; 2 BioTekna®, 30020 Marcon-Venice, Italy; dario.boschiero@biotekna.com; 3 Institute for Successful Longevity, Florida State University, Tallahassee, FL 32306, USA; jilichernst@fsu.edu

**Keywords:** osteosarcopenic obesity, visceral fat, nutrition, exercise, treatment

## Abstract

Osteosarcopenic obesity (OSO) syndrome describes the simultaneous deterioration of bone, muscle and excess fat, resulting in reduced functionality and systemic metabolic dysregulation. The key component contributing to this may be ectopic fat in the viscera, bone and muscle. OSO research to date is summarized, and the revised criteria for its identification for research purposes are reviewed and proposed, including new criteria to assess visceral fat in males and females. Finally, nutritional and physical activity recommendations are consolidated into a treatment algorithm, which can be validated in future studies and which may also be applied to preventative management.

## 1. Introduction

The original osteosarcopenic obesity (OSO) concept paper outlining the whole body and cellular connections among the three tissues, bone, muscle and fat, was published in 2014 along with the preliminary diagnostic criteria [1]. More refined diagnoses and some preliminary treatment modalities followed in subsequent papers [2,3]. One of the key elements of OSO is the linked nature of the syndrome—from its cellular connections to the deterioration of bone (osteopenia/osteoporosis), muscle (sarcopenia) and excess adipose tissue (overweight/obesity, including the redistribution of fat into the visceral area and adipogenesis in bone and muscle tissues) [1]. Osteopenia/osteoporosis, sarcopenia and overweight/obesity were once considered as separate conditions and were rarely studied together. The combination of two tissues, osteoporosis and sarcopenia [4,5] or sarcopenia and obesity [6,7,8], has recently been discussed. It was rarely a case of all three tissues together (OSO), apart from preliminary suggestions that conditions of the bone, muscle and fat are linked [9,10]. Recognizing the coexistence (comorbidity) and, possibly, the co-development of conditions/diseases may be more physiologically relevant and may help guide an improved treatment plan as opposed to treating each single disease state as it were a separate entity. We have already discussed the distinction between the physical and functional characteristics of OSO [2], and we are not proposing any revisions to the latter. This paper focuses on the physical diagnosis of OSO, using dual-energy x-ray absorptiometry (DXA) or other technologies, in males and females and especially in the context of ectopic fat.

Our objectives in the present review are (a) to provide an update on the current knowledge of OSO from the time it was first presented as a new syndrome in the form of a short review of the papers published so far; (b) to point out the current difficulties with the physical diagnosis of OSO and to present an improved diagnostic criteria that includes males; and (c) to provide consolidated treatment modalities, both nutritional and physical activity, suitable particularly for mid-age to older individuals with OSO.

## 2. Literature Search Methods

This review was primarily narrative in nature. Literature searches utilized PubMed but also included government and professional/health organizations, Google Scholar (without patents and citations) and article reference lists. Peer-reviewed articles published between 2013 and 2019, articles related to sarcopenic obesity, and review papers that summarized relevant data (e.g., cutoffs) were included. Individual authors first screened the results using the abstract and followed up with the article to ensure the pertinent data were present.

The literature searches focused on the following areas:

1. osteosarcopenic obesity
2. definitions and cutoffs for sarcopenia, obesity and visceral fat

A literature review of research specific to OSO was not performed previously to our knowledge; therefore, only the phrase “osteosarcopenic obesity” was used to capture any articles with an exact match. In addition, we did not want to limit the search as we anticipated a relatively low number of results. These articles that directly measured/studied OSO formed the basis for Section 3 and Section 7.

For Section 4, Section 5 and Section 6, literature searches were performed using combinations of words and/or phrases, as well as synonyms, including “bone [mineral]”, “trabecular”, “cortical”, “density”, “sarcopenia”, “obesity”, “fat”, “visceral fat”, “subcutaneous fat”, “definition”, “cutoff”, “prevalence”, “body mass index”, “body fat [percentage]”, “bone mass”, “lean mass”, “fat free mass”, “android fat”, “gynoid fat”, “imaging”, “measurement”, “serum”, “biomarker”, and “bioelectrical impedance”.

## 3. State of the Current Knowledge

**Figure 1:**
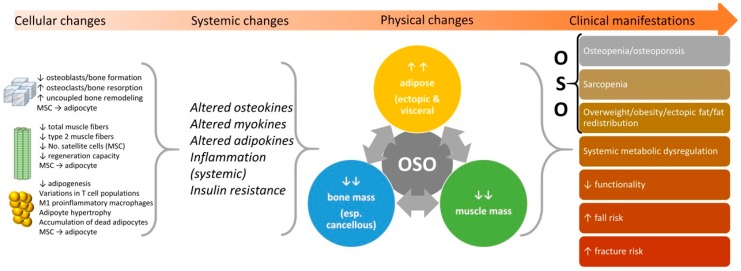
The progress of osteosarcopenic obesity (OSO) from cellular changes to clinical manifestations. Adapted from References [1,13,14,15,16,17,18]. No need for copyright.

OSO is progressive, and it can begin with any one of the three conditions: osteopenia/osteoporosis, sarcopenia or obesity/visceral fat/fat redistribution, each originating on cellular and endocrine levels, starting from the stem cell lineage commitment deregulation and changes in bone, muscle and fat cross talk via alterations in osteokine, myokine and adipokine concentrations, respectively [1]. Each condition if left untreated can culminate into the triad of bone, muscle and fat deterioration or OSO syndrome [1,2], leading to the elevated risks for immobility, falls, fractures and disability [11,12] (see Figure 1), and probably an array of other chronic diseases that have yet to be determined and learned about.

Since the publication of our original concept paper [1], we expanded our own research, as outlined below, while other research groups also studied OSO [19,20,21,22,23], with various results for its prevalence and connections with different comorbidities. In some studies, OSO was not specifically identified, but from the characteristics of the population exhibiting osteopenia, sarcopenia and increased adiposity, it was clear that the OSO phenotype was present [24,25].

Epidemiological data from the Korean National Health and Nutrition Examination Surveys showed that individuals ≥50 years with OSO had lower serum vitamin D levels [21], lower diet quality [20] and higher dietary inflammatory index [26] compared to their counterparts without OSO. While low-grade chronic inflammation has been linked to the development of OSO [1], a recent review shows the association of chronic stress, including dehydration, with OSO [27]. A study in Chinese older women showed that those with OSO had a more unfavorable blood lipid profile [22] compared to those without OSO while Mexican middle-age and older women with OSO had a significantly lower physical performance and poorer frailty scores [12]. However, the most striking finding was the presence of OSO in apparently healthy young overweight/obese Greek males and females compared to their normal weight counterparts [28] as the OSO phenotype is not anticipated in young healthy individuals.

A previous study showed the link between OSO and a lower handgrip strength, a poorer balance and inferior walking abilities in older women compared to age-matched obese-only women [11]. In addition to this lower functionality, the intrinsic complex nature of the OSO syndrome may increase the risk for falls [19]. Based on fracture site, OSO prevalence was more common in the vertebral, hip and ankle fracture groups for men, but for women, OSO prevalence was only higher in the ankle fracture group [29]. In 543 Mexican males and females, OSO (16.6% of study population) was independently associated with frailty (as measured by the Frailty Phenotype and FRAIL scale) and a lower Short Physical Performance battery score [12]. Perna and colleagues divided OSO into osteosarcopenic visceral obesity (OSVO) and osteosarcopenic subcutaneous obesity (OSSO) [23]. While the overall prevalence of OSO was 6.79% in 801 bedridden hospital patients 65 years or older, OSVO was more frequent (4.56%) compared to OSSO (2.22%) and was associated with worse metabolic health, inflammation and a higher risk of fractures. This suggests visceral obesity, or ectopic obesity, may be more common in OSO.

A study in Korean middle aged and elderly males and females, 50 years or older, found that sarcopenic obesity was strongly associated with osteoporosis [30]. Body mass index (BMI) was used to classify obesity, and the sarcopenic obese group (SO) had the highest percent of body fat for both males (32.8%) and females (43.4%) compared to the other groups. However, their data also show that OSO was present, although the authors did not specifically refer to it as a separate entity. Using their unweighted sample size data, of the 150 males with SO, 64 had osteopenia and 41 had osteoporosis, showing 105 males (70% of SO group) with OSO. For females in the SO group, 67 had osteopenia and 121 had osteoporosis from 201, indicating 188 (93.5%) had OSO. For OSO, this is a prevalence of 3.1% for males and 5.4% for females, or a total prevalence of 4.2%, from a total sample size of 3385 men and women.

More evidence is accumulating which associates obesity to both lower bone and muscle mass. In a study of self-reported fractures over 10 years in sarcopenic obese men and women, the protective effect of fat mass on bone was negated by the presence of sarcopenia [31], suggesting an interdependent link among three tissues. The dysmobility syndrome in the Osteoporotic Fractures in Men MrOS cohort [24] and others [32,33] differ somewhat in criteria but are similar in concept. For MrOS, the diagnostic criteria included appendicular lean mass/height^2^ (<7.26 kg/m^2^), total body fat (>30%), spine or hip T-score (≤−2.5), grip strength (<30 kg), gait speed (<1.0 m/s) and falls (≥1 in 12 months) [24]. Each factor was scored 1 or 0, and a score of ≥3 indicated dysmobility syndrome. Eight percent (8%) of the MrOS cohort was diagnosed with dysmobility syndrome, which was strongly associated with major osteoporotic fracture. As bone, muscle and fat mass cutoffs are included in dysmobility syndrome, this suggests that OSO is indirectly measured in males. Another study (where the article was in Japanese, but the abstract was in English) found that the prevalence of sarcopenic obesity was 16% among older Japanese women (mean age 72.5 years) [25], however, over half of these women with sarcopenic obesity had osteopenia (defined as 20% below the reference), which was in effect a diagnosis of OSO.

In the Busselton Healthy Ageing Study (Australia), a higher body fat was associated with a lower bone mineral density in females but not in males; however, the highest body fat groups also had the lowest lean mass index (kg/m^2^) in males and females [34], alluding to the presence of OSO. The Louisiana Osteoporosis Study, a large cross-sectional study of those 18 years or older, showed a total prevalence of sarcopenic obesity of 7% in males and 2.5% in females, with the older age groups contributing the most [35]; this study also has bone density data, making it an ideal database to estimate the prevalence of OSO.

The microbiome is increasingly associated with various conditions, and there is some early evidence that links an altered gut microflora to osteoporosis [36,37], sarcopenia [38] and obesity [39]. By evaluating the dietary changes occurring with aging, especially in long-term care settings, we found that these changes may contribute to the less favorable microbiota and place elderly at the higher risk for OSO [40].

OSO has not been explicitly investigated in disease states; however, Carsote and colleagues suggest that malignancy might lead to OSO and that malignancy in the presence of OSO would result in a worse prognosis [41]. While not reported as OSO, in males with or without HIV, all three components, osteoporosis (femoral neck T-score), sarcopenia (appendicular lean mass/height^2^), and obesity (visceral adipose tissue area), were associated with frailty but not BMI or subcutaneous fat [42]. The authors emphasize that interventions for all three conditions (OSO) would help to treat and reduce the risk for frailty. In a sample of 40 women with rheumatoid arthritis (RA), with a mean age of 40.7 years, 17 (42.5%) were diagnosed with OSO [43], suggesting RA may promote the development of OSO or vice versa. A case study in identical twins 76 years of age suggested that the twin undergoing androgen deprivation therapy for prostate cancer is at higher risk for OSO and frailty due to the overt body composition changes [44].

Taken together, there is a growing body of evidence to support the existence of OSO in women and men and the role of fat mass in bone and muscle loss with aging. Even more important is that many databases are still available (based on numerous published articles) which could be used to retrospectively estimate the prevalence of OSO (this was one of our objectives in the concept paper; see Reference [1]), which would help to further define and characterize the syndrome.

## 4. Persisting Difficulties in the Identification of OSO and Its Components

OSO’s current physical diagnostic criteria lead to its identification when it has reached clinically relevant stages for bone loss, muscle loss and fat mass gain. Briefly, in our previous paper [2], we outlined two options for diagnosing OSO: by physical measurements of bone and body composition via DXA and/or via functional performance measures as a proxy for bone and body composition data, when the former latter are not available. When both measurements (bone and body composition and functional performance) are available, they can provide a more comprehensive way to grade the extent of the physical and functional deterioration and diagnose OSO syndrome (see Reference [2]). However, our previous criteria, for women only, did not fully consider the role of ectopic fat in OSO. Furthermore, the development of alternative technologies could make OSO identification even easier, as reviewed below.

### 4.1. Bone and Muscle Tissues

The definitions and cutoffs for osteopenia and osteoporosis are well-established. Although not perfect, they are based on the extensive research in the last several decades and are widely used in the medical community [45]. A position statement from the National Bone Health Alliance Working Group [46] suggest that the diagnosis of osteoporosis should consist of a low T-score combined with a high risk of fracture, assessed by the Fracture Risk Assessment Tool (FRAX^®^) tool [47]. The limitations of DXA for the routine screening and diagnosis of osteoporosis are its availability and resource needs. However, bone mass measurements using bioelectrical impedance are becoming more commonplace. The instrument used to detect OSO in a young population [28], BIA-ACC^®^ (BioTekna srl, Marcon-Venice, Italy), includes bone mass (kg; whole body) as well as other body composition parameters and has been developed and validated against DXA [48]. This instrument gives the total quantity of bone in the body but cannot distinguish among different skeletal sites like DXA. The bone status is determined based on a normal reference population (which may be instrument specific); therefore, a T-score of ≤−1 SD would indicate a lower bone mass (osteopenia/osteoporosis). However, current approaches to defining osteopenia/osteoporosis remain rooted in bone mass and do not account for changes in bone marrow fat, trabecular changes [49] or ectopic fat in bone. Baumgartner and colleagues were one of the first groups to propose a criterion for sarcopenia using DXA in normal-weight individuals [50]. DXA and Bioelectrical Impedance Analysis (BIA) are used to measure muscle mass with the caveat that neither technology identifies muscle but the total lean mass (sum of muscle, total body water and organs), which is used as a proxy for muscle. This limitation is offset by including only the lean tissue from the arms and legs (appendicular) which mostly comprise of muscle [51]. However, the presence of fat mass and ectopic fat mass may also complicate this approach.

To address this issue, Baumgartner et al. suggested that reliable accurate instruments, such as DXA, be used for the simultaneous assessment of sarcopenia and obesity [52], suggesting a single device is better than several to determine combinations of conditions. This subsequently led to the identification of sarcopenic obesity as a separate entity [53,54]. The criteria used to define sarcopenia in the presence of obesity are heterogeneous, although skeletal mass index (SMI) seems to be the most common method, but with different cut points used by researchers (ranged from <7.23–8.81 kg/m^2^ for men and <4.70–7.36 kg/m^2^ for women) [55]. Sarcopenic obesity for women could be determined from the Domiciano et al. (2012) equation [56], derived from the negative residuals of the linear regression model in which appendicular lean mass (ALM) is adjusted for fat mass and height and in which the 20th percentile was defined as the cutoff point for sarcopenic obesity [2,56]. For men, the original Baumgartner et al. definition was found to correlate too closely with BMI in the Health, Aging and Body Composition (Health ABC) study, and was believed to underreport sarcopenic obese [57]. For sarcopenia itself, the Foundation for the National Institutes of Health (FNIH) determined the ALM/BMI ratio cutoff values of <0.789 and <0.512 and handgrip strength cutoffs of <26 kg and <16 kg for both older men and women respectively [58]. The percent fat mass seems to be the most common option for the diagnosis of sarcopenic obesity in different populations, with cutoff values ranging from >20.21–28.00% body fat for men and >31.71–42.90% body fat for women [55].

### 4.2. Obesity Classification and Adipose Tissue

Apparently, one of the still most-debated aspects in body composition evaluation, in general and in the physiological sense as it relates to OSO, is the assessment of obesity [59,60]. Even more important in this context is the inability to assess visceral fat or the infiltrated fat into bones and muscle (ectopic fat), which will be discussed later. The most common way of classifying overweight/obesity in the clinical setting is still by calculating BMI, despite its shortfalls [61,62]. The story of the development of BMI is colorful, and there are still a number of studies to support its utility [63]. Measuring BMI is useful if the outcome is to change BMI, which is really a change in weight. Furthermore, a low BMI (underweight) is also associated with sarcopenia (especially in older adults) [64], osteopenia/osteoporosis [65,66,67] and frailty [68], the latter exhibiting reverse epidemiology with body weight [69]. This supports the notion of normal-weight obesity [70] and suggests that the location of fat mass and the overall volume of lean mass are more important than body weight. Therefore, in the context of OSO, where visceral and/or infiltrated fat is/are the target(s), weight or BMI would not be useful for identifying the status of obesity or as outcome measures.

There has been a refinement of BMI by dividing it into the fat mass index (FMI) and fat-free mass index (FFMI). These are essentially components of BMI, are mathematically FMI + FFMI = BMI and could be utilized to better describe the amount of lean and fat mass within a given BMI [54]. FMI increased up to age 65 in women and age 80 in men in data from the National Health and Nutrition Examination Survey (NHANES) [71]; however, a Swiss study showed a continual increase in FMI in males and females aged 35 years or older [54]. From the NHANES data, FMI cutoffs to diagnose obesity are >9–12 kg/m^2^ for males (Obese Class I, or BMI > 30 kg/m^2^) and >13–17 kg/m^2^ for females (Obese Class I, or BMI > 30 kg/m^2^) [71]. Obese Class II and II cutoffs are also present, but a BMI > 30 kg/m^2^ is sufficient to diagnose obesity. FMI cutoffs derived by Schutz and colleagues using bioelectrical impedance in a Swiss study are 8.3 kg/m^2^ for males and 11.8 kg/m^2^ for females [54,72], representing a BMI ≥ 30 kg/m^2^.

It becomes obvious that a more unified definition of obesity is required to reflect the negative impact of excess fat mass on metabolic and organ health, which still might not be available. The Framingham Study used obesity cutoffs of >30% for men and >40% of body fat for women [73], although the mean body fat percentages for males and females were 27.5% and 39.3%, respectively. The obesity cutoffs that are usually stated as the criteria of the World Health Organization (WHO) (≥25% body fat for men and ≥35% body fat for women) may be misquoted [74], meaning official WHO body fat percentage cutoffs to define obesity do not exist. The body fat percentage chart from the American Council on Exercise has cutoffs for obesity of 25% and 32% for males and females, respectively [75], while the American College of Sports Medicine have obesity cutoffs by age group and sex, which for 60 years or older is 28.5% for men and 36.6% for women [76]. Using data from 3327 participants (62% female) in the U.S., the reference ranges for body fat percent were developed [77]. For males, 25% body fat corresponds to the 80th percentile for 60–79-year-olds in the reference range, and 35% body fat was found in the 80th percentile of females 60–79 years of age. The highest percent body fat was seen in males 40–49 years of age in the 10th percentile (41.0% body fat), and the highest percent body fat for females (52.2%) was also in the 10th percentile but in those 60–69 years of age. In addition to the different cutoffs and the lack of a consensus as to which particular ones to use, no measures or cutoff reviewed above consider the inevitable redistribution of fat into the visceral area and its infiltration into bone and muscle with age (causing much more metabolic derangements than subcutaneous fat) [78].

As we outlined previously [79] and as it is supported by recent evidence [23], the altered adiposity that needs to be present to meet the criteria for identification of OSO, besides the overt excess of adipose tissue (overweight/obesity), includes the redistributed fat around visceral organs. In addition, the altered adiposity, even without overt overweight/obesity, could occur when fat infiltrates into bone and muscle, creating fatty bones and muscles (first recognized by Rosen et al., [80]). This derangement of adipose tissue is one of the key contributors to the simultaneous loss of bone and muscle, ultimately leading to clinical manifestations, such as frailty, in addition to other metabolic impairments (e.g., metabolic syndrome or cardiovascular disease). Visceral fat is the site of pro-inflammatory cytokines secretion [81], and this contributes to metabolic imbalances. Visceral fat and adipogenesesis in bone and muscle increase steadily with age, and there are many contributing factors, including overall fat mass gain and muscle loss, as well as a decreased metabolic rate and age-related hypogonadism [60,78].

Regarding visceral fat cutoffs, based on the risk of metabolic disease, Hunter and colleagues suggest conservative cutoffs of 130 cm^2^ for men and 110 cm^2^ for women by Computerized Tomography (CT) or Magnetic Resonance Imaging (MR) of the abdominal region with the expectation that men and women would reach these cutoffs in their 5th and 6th decade of life, respectively [78]. Another method to measure visceral fat (visceral obesity), using DXA data transformed to CT data, is the visceral to subcutaneous fat ratio, with a value of >1 indicating visceral obesity [23]. Since visceral fat is mostly a component of central or abdominal fat, a simple proxy for visceral fat may be waist circumference [78,82,83]. Furthermore, the link between waist circumference and metabolic derangements is firmly established as it is a National Cholesterol Education Program (NCEP) Adult Treatment Panel III (ATP III) criterion for the diagnosis of Metabolic Syndrome, with cutoffs of ≥102 cm (≥40 inches) for men and ≥88 cm (≥35 inches) for women [84]. An alternative to waist circumference is the waist–hip ratio (W/H ratio), which in 1998, was included in the World Health Organization’s criteria for Metabolic Syndrome, with cutoffs of >0.90 for men and >0.85 for women [84,85]. It must be noted that only the WHO criteria include the W/H ratio; however, this measure is also limited since the W/H ratio determines outside circumferences and not necessarily internal fat measures [86]. Regardless, waist circumference and the W/H ratio are rapid and inexpensive measures that can be performed in any setting and in a wide variety of individuals, which may ultimately serve as measures of risk (screening tool) for the presence of visceral fat.

Another alternative to measuring visceral fat is to calculate android and gynoid fat, made possible by newer DXA instruments which measure/analyze the android (abdominal) and gynoid (hip) fat regions of the body [87]. Visceral fat correlates better with android than with gynoid fat, and this may be explained by the much lower presence of gynoid fat in males [88]. However, the android to gynoid fat ratio (A/G ratio) is also a useful measure as it is a good predictor of metabolic derangements and cardiovascular disease in adults [89,90] and children [91], similar to visceral fat. An A/G ratio <1.0 is what is normally recommended, although, when creating reference standards, Imboden et al. found that males 60–69 and 70–79 years of age had 10th percentile values of 1.01 and 1.15 respectively [77]. However, we still included the A/G ratio of ≤1 for both males and females in our updated criteria for OSO. In cases when DXA is not available; a proxy for the A/G ratio could be waist circumference or the W/H ratio, as discussed above.

## 5. Updated OSO and Ectopic Fat Identification

Based on the data reviewed, we propose here new and improved methods of identifying OSO using visceral adipose tissue to account for the fat redistribution into the abdominal area (occurring inevitably with age) and expanding the criteria to both men and women, presented in Table 1. The criteria for osteopenia and osteoporosis are well-established and have not changed for males and females. The cutoff for OSO is set at a T-score ≤ −1 standard deviation (SD) below the reference to include both osteopenia and osteoporosis (the latter defined as −2.5 SD), using DXA. In view of the use of bioelectrical impedance to measure bone mass, we propose a bone mass (kg) T-score ≤ −1 SD below the reference for BIA-ACC^®^. For muscle mass (sarcopenia), the cutoff values for males were added to our original OSO criteria [1,2]; however, the criteria for females remain unchanged. A criterion was added, specific to bioelectrical impedance instruments, based on OSO data for BIA-ACC^®^; for low muscle mass, we propose an S-Score of ≤−1.0 SD below the reference. Fat mass and the fat mass index are included to measure obesity, while waist circumference, visceral fat, visceral/subcutaneous fat ratio, intramuscular adipose tissue (IMAT) by BIA-ACC^®^ and the A/G ratio are used to determine the extent of abdominal/visceral fat. BIA-ACC^®^ cutoffs were included as it has been used to identify OSO [28]; however, there may be other bioelectrical impedance instrument specific reference values. As a final point, in addition to CT and MRI to measure visceral fat, DXA methodology is available, briefly described here [92], and has been shown to correlate well with CT data [92,93,94], which expands the role of DXA in identifying OSO.

We envision a grading method based on some of these measures in the future; however, not enough data on OSO are available currently, and more research is needed. We did not include BMI as a preferred method to define obesity in the context of OSO for two reasons; (1) it does not indicate body composition and (2) it would exclude normal-weight obesity, which may be at risk for OSO. However, in the absence of other measures, BMI, with appropriate ethnic cutoffs, may suffice to obtain an initial diagnosis of obesity, if that is the research area of interest. It is important that visceral fat and other body composition parameters are measured so the extent of the metabolic burden of obesity (systemic and on bone and muscle) can be determined. Although the preference is to use DXA, BIA and, whenever possible, CT or magnetic resonance imaging (MRI) to measure ectopic fat mass and to identify OSO, we have included proxy measures of ectopic fat to be easily used in the field or clinical setting. Finally, we have utilized broadly established cutoffs or those of European origin; however, we encourage adjustments to the criteria for various ethnic groups.

## 6. Future Refinements for OSO Diagnosis and Treatment

While our original recommendations were always meant to be improved upon, by us and/or other researchers as previously discussed [3], there are still some work to be done to achieve a complete and most comprehensive diagnosis of OSO syndrome, originating from the lack of consensus and/or inadequate technology for some aspects of body composition assessment (e.g., obesity, infiltrated fat, sarcopenic obesity and osteopenic obesity). It was suggested that newer technologies, such as quantitative computed tomography, EchoMRI (Quantitative Magnetic Resonance), OsteoProbe and ultrasonography, be utilized for the diagnosis and management of OSO [59]. It needs to be noted that, in some other published studies related to OSO (as reviewed above), devices other than DXA were used for its identification, such as bioelectrical impedance [28]. While the gold standard measure may be debated, it is acceptable to use other instruments, especially on a longitudinal basis to measure changes to body composition over time while OSO is treated. There is still no convenient tool to assess infiltrated fat into bone and muscle other than magnetic resonance imaging (MRI) which is still not suitable for routine general screening. However, there is an opportunity to add the diagnosis of osteopenia/osteoporosis, sarcopenia and obesity (especially visceral fat), or OSO, to scheduled abdominal CT/MRI scans [95], which ultimately may help with treatment plans and improve outcomes.

Although OSO combines all three conditions (osteoporosis, sarcopenia and obesity) into one syndrome, combinations of two conditions are also possible. The identification of osteopenic obesity and sarcopenic obesity are still in development as these two entities, especially the former, are just beginning to be recognized by the scientific community [1,2,6,8]. Therefore, while the revised diagnostic criteria in Table 1 are meant for the identification of OSO, they allow for the diagnosis of single or dual conditions.

Presently, there are technologies available that could be utilized and further developed for the future diagnosis of OSO and its components. However, there needs to be a concerted effort to develop devices that can be used in the field/clinic as well as in a research setting to accurately assess visceral fat and other body composition compartments, so more studies can be done to refine the diagnostic criteria for bone and muscle loss and fat mass gain/redistribution. Some of these are addressed in the next section.

### 6.1. Bone

A portable, newly developed and validated device, OsteoProbe (ActiveLife Scientific Inc, Santa Barbara, CA, USA), could be utilized for the analysis of bone strength and quality. OsteoProbe measures reference point micro-indentation to determine the bone’s ability to resist the separation of mineralized collagen fibrils. The output is a bone material strength index (BMSi)—a ratio of the indentation distance in bone versus a reference material [96,97]. The micro-indentation enables the further identification of bone fragility and quality independently of BMD (which provides only areal bone mineral density) [98]. Thus, combining BMD, T-scores and BMSi in addition to the percentage body fat or visceral fat could provide a better assessment of bone health status.

Measuring bone parameters using BIA is no longer the exception, probably due to its convenience. BIA-ACC^®^ (BioTekna^®^, Marcon-Venice, Italy) was recently validated against DXA [48] for the determination of total bone mass (kg). However, while this instrument is versatile in relation to OSO, it provides whole body bone mass and a T-score, it cannot distinguish among different skeletal sites.

MRIs can assess the amount of bone marrow fat, which would give further insight into fat infiltration in bones. In older women, there is a corresponding increase in vertebral-bone fat, reflected as yellow bone marrow, with declines in BMD [99,100,101]. Interestingly, bone marrow fat is reduced with pharmacological osteoporosis treatments [102]. Even the type of marrow fat may have an effect on BMD, with higher saturated fatty acid concentrations measured by magnetic resonance spectroscopy (MRS) and associated with osteopenia and osteoporosis [102]. A recent study also found femoral BMD to be correlated with total femoral marrow fat, probably signaling osteoporosis [103]. Yellow or fatty bone marrow may further aggravate bone loss and frailty, as adipocytes compete with osteoblasts in bone marrow (both originate from the same precursor), suggesting that an increase in marrow fat may actually promote bone loss [101]. In addition, an increase in marrow fat appears to be a biomechanical compensation for trabecular thinning [99]. Currently, MRI is the only clinical imaging technique that allows the direct visualization of bone marrow with a high spatial resolution [104]. Considering the impact of bone marrow fat on bone strength and structure, MRI scans would be the ultimate tools to provide validation for any other device in development.

There is also the opportunity for current technologies (CT and DXA) to evolve better measures of bone status through higher resolutions and new algorithms and for bioelectrical impedance to develop site-specific (trunk, leg, etc.) measures using new sensor technologies, different frequencies or new algorithms.

### 6.2. Muscle

Measures such as muscle quality may become more important in the future as treatments for sarcopenia alone, OSO or other combinations of conditions become more commonplace. Muscle quality reflects the overall tissue health and could be estimated by calculating the phase-angle from the BIA data by finding the arc tangent of the reactance to resistance ratio (arc-tangent reactance/resistance × 180°/π) [105]. Although not directly measuring the infiltrated fat in muscle, the phase angle could serve as a proxy for the estimation of the quality of the muscle tissue. Similarly, some researchers are using the knee extension measurement to assess muscle quality, as well as lower body strength, where one repetition maximum (RM) in kg from the knee extension is divided by the leg lean mass in kg (measured by DXA or BIA) [106,107]. Muscle quality can be assessed with CT, providing skeletal muscle density (SMD) and intramuscular adipose tissue (IMAT) [108]. SMD is based on a radiological scale of tissue density in Hounsfield Units [109], while IMAT measures visible adipose tissue within muscles in cm^2^, using predetermined muscle and fat density cutoffs. Interestingly, IMAT cutoffs associated with hospital mortality exist and are <170 cm^2^ and <110 cm^2^ for critically ill male and female patients, respectively [108]. Alternatively, muscle quality can be estimated by ultrasound devices. High-frequency ultrasound precisely measures skeletal muscle mass, muscle length and density and can even track muscle atrophy [110,111]. An ultrasound probe can evaluate the quality of skeletal muscle, such as the quadriceps, by finding the echo intensity using gray scale analysis with software such as Adobe Photoshop [112]. Echo intensity is negatively correlated with muscle strength, muscle thickness and age [112]. Finding the echo intensity from ultrasound scans is also useful in identifying myosteatosis (infiltrated fat) and the aging muscle. MRI accurately measures the myofibril cross-sectional area and quadricep volume and is able to detect small differences in muscle hypertrophy or atrophy [113,114], and this can provide a comprehensive assessment of muscle, including fat infiltration, better than from DXA or BIA although access to MRIs and CTs for body composition are not routine. As indicated by Lenchik and Boutin (2018), the automated diagnosis of osteoporosis, sarcopenia and obesity from CT data through machine learning would greatly reduce the resources required for CT analysis and possibly improve access and mainstream use [115].

Relatively newly developed portable devices based on multifrequency bioelectrical impedance are BIA-ACC^®^ and PPG-StressFlow^®^ (BioTekna^®^, Marcon-Venice, Italy). BIA-ACC^®^ assesses quantitative and qualitative body composition data and contains information on total body water, extracellular and intracellular water, fat mass and fat free mass (kg and % body weight), total bone mass (kg) and T-score, skeletal muscle mass (kg and % fat free mass) and S-score as well as IMAT. BIA-ACC^®^ was validated against DXA in postmenopausal women of varied weight and body composition [48]. PPG-StressFlow^®^ provides analyses, monitoring and biofeedback of the autonomic nervous system and heart rate and providing information on chronic stress, systemic inflammation, oxidative stress and insulin resistance [27,116]. Apart from their portability and ease of use, combining BIA-ACC^®^ and PPG-StressFlow^®^ will not only provide a body composition assessment (bone, muscle and fat) but also show underlying metabolic derangements such as underlying low-grade chronic inflammation and stress, offering a more comprehensive assessment of an overall health status.

Overall, while further studies are needed to determine the exact cutoffs for the various instruments and to validate them in order to be included into the OSO diagnostic criteria, it may be more important to begin assessing sarcopenia and ectopic adiposity with whatever tools are available.

### 6.3. Serum Markers of OSO

Serum markers of bone turnover, muscle functioning and inflammation could be used as complementary tests to further develop/confirm the diagnostic criteria for OSO and to give more insight into the quality and changes in any of the tissues. For example, osteocalcin and carboxy-terminal collagen crosslinks (CTx) are well-known and often-used markers to indicate an imbalance in bone turnover and the deterioration of bone tissue [1]. Furthermore, osteocalcin has an inverse relationship with visceral fat and pro-inflammatory markers [117,118]. Serum sclerostin, an inhibitor of bone formation, and skeletal muscle specific troponin T (sTnT), a marker for muscle turnover, were significantly higher in those with OSO and osteopenic obesity compared to obese only [119], suggesting OSO may be predicted using a combination of biomarkers. The search for biomarkers may be accelerated using the ovariectomised rat, which is a potential model for OSO research in women [120]; however, an animal model for male OSO will need to be developed and could include hypoandrogenic induced animals. Serum sTnT has been recently associated with the onset of sarcopenia, and it dropped proportionally with improvements in handgrip strength in older adults [121]. Therefore, assessing markers of sarcopenia to complement the measurements of muscle mass and quality (DXA, BIA and ultrasound) would add to the specificity of diagnosis.

With increased adiposity and visceral fat accumulation, circulating levels of inflammatory cytokines such as TNF-α, IL-1 and IL-6 and C-reactive protein are higher, signaling chronic inflammation [122,123]. A recent review presented mechanisms of chronic low-grade inflammation causing and/or perpetuating both obesity and osteoporosis [81]. Furthermore, inflammatory cytokines TNF-α and IL-6 and C-reactive protein are also elevated with sarcopenia and sarcopenic obesity [124,125] and in individuals with reduced handgrip strength [125,126]. To tie this together, as bone and muscle share genetic and endocrine influences, a low muscle mass has a negative impact on BMD, especially in the femoral neck [127], while adipose tissue perpetuates inflammation and, by infiltrating into bone and muscle, compromises their integrity and function. Therefore, it is conceivable to assume that inflammatory markers secondary to obesity and/or sarcopenia also damage muscle and bone and could be examined when evaluating OSO.

## 7. Nutritional and Physical Activity Considerations for OSO

### 7.1. Nutrition

Expert nutritional and other intervention guidelines with varying levels of evidence exist for the prevention and/or treatment of osteoporosis, sarcopenia and obesity (for example, References [45,128,129]), as well as sarcopenic obesity [53]. OSO was described as being a multifaceted syndrome; therefore nutritional, exercise and pharmacological interventions would be required [19]. Guidelines for the management of OSO are now available [3,19,59,60,130,131,132]. One of our focus areas over the last five years was on the nutritional aspects related to OSO [3,60,131,132,133,134]. Our intention here is to compile and create preliminary treatment principles for the management of OSO. Treatment plans for OSO will require lifestyle intervention (nutrition and physical activity) and possibly pharmacotherapy, and will probably require a multidisciplinary team, including physicians, dietitians and physical therapists.

Reduced energy and protein intake, the overconsumption of high glycemic carbohydrates and low intakes of n-3 polyunsaturated fatty acids [132] may all contribute to OSO. A chronic high saturated fat and sucrose diet led to the deterioration of bone and muscle and obesity in mice [135], supporting the premise that a poor diet contributes to OSO. Insufficiencies in calcium; magnesium; potassium; vitamins B_6_, B_12_, A, D, E and K; and folate, as well as excesses of sodium, phosphorus and most B vitamins (with regard to the Dietary Reference Intakes) may be contributing to OSO [131]. However, a more recent concept of nutrient ratios may better to help explain how the overall nutrient insufficiencies and excesses contribute to OSO and overall health [133,134].

Higher protein intakes for the management of OSO are universal within the guidelines for OSO, with good reason, as the benefits of more protein are well-known for both bone [136,137,138] and muscle [136,139,140,141,142,143]. Higher vitamin D and protein are seen as important interventions for the South Korean ageing population to prevent OSO [144]. There are no specific requirements for total carbohydrate for OSO, although a better dietary carbohydrate quality, including more fiber and less simple sugars and/or high glycemic index foods, is recommended [3,132,134]. Similarly, there is no recommendation for total fat intake; however, increasing the intake of the n-3 polyunsaturated fatty acids, eicosapentaenoic acid (EPA) and docosahexaenoic acid (DHA) to more than 1 g/day and the intake of the essential fatty acid α-linolenic acid to more than 1 g/day is suggested [3,130]. The primary vitamin that is advised to be consumed in a greater quantity is vitamin D; its role in bone, muscle and fat health is well-established, while minerals, calcium, magnesium and potassium are the most critical [3,130]. Aside from the nutrients mentioned above, it is probably important to target obtaining the Dietary Reference Intakes for all nutrients as part of an overall healthy dietary pattern. Overall, the nutritional guidelines are designed to address insufficiencies in dietary intake; however, the insufficiencies combined with a poor carbohydrate quality and probably insufficient n-3 PUFA intakes can create an unfavorable nutritional environment that may ultimately promote OSO [131,132,133,134].

### 7.2. Physical Activity

**Figure 2:**
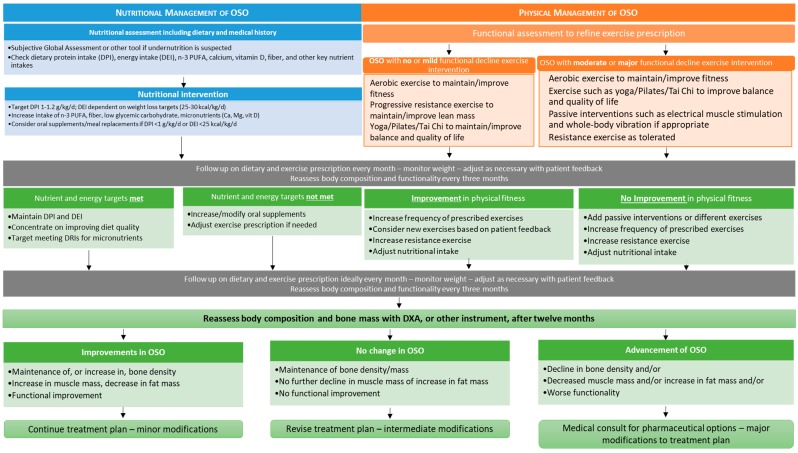
The nutritional and exercise treatment principles for OSO.

Resistance training has been directly shown to improve muscle mass and strength and reduce body fat in older Brazilian women with OSO, although, as expected, there was no improvement in bone density over 12 weeks [145]. A sedentary lifestyle over time can contribute to OSO development [18], so exercise is an important intervention for OSO treatment and management. If frailty is present [12], the immediate goals could start with alternative exercises such as yoga, Pilates or Tai Chi, or just walking could be employed to improve balance, flexibility and strength in place of resistance or strength training [146]. The various guidelines agree on the need for a variety of exercises as OSO is a complex entity [3,19,130,146]. However, in extreme situations, whole body vibration or electrical muscle stimulation could be utilized in place of standard resistance training or alternative exercises [146]. Overall, the exercise interventions are designed to maintain bone, to build strength, to lose fat mass (especially visceral) and to improve balance (prevent falls) and quality of life. Figure 2 contains the preliminary guidelines for the management of OSO, compiled from References [3,19,59,60,130,131,132]. This algorithm combines nutrient and exercise recommendations as diet and exercise are intricately linked.

Figure 2 shows that the nutritional management begins with a full assessment if undernutrition is suspected or to rule it out. In addition, an assessment of nutrient intake from foods and supplements should be performed to form a baseline for nutritional intervention. Nutritional intervention begins with an initial protein target of 1–1.2 g/kg/day. While higher intakes have been suggested [3,130], it may be more beneficial if the requested dietary change is performed in smaller steps. Protein and other nutrient intakes can be individualized depending on progress and adherence. Other key nutrients should be increased, through diet and supplements if necessary. A follow-up should be scheduled for one month after beginning the intervention to assess for dietary adherence.

Physical management begins with a functional assessment, assuming OSO was diagnosed using DXA or another instrument. The functional assessment scoring has been previously described [2], and from the established baseline, it helps to determine which exercises are suitable. A follow-up should be scheduled one month later to assess physical and functional progress. After 3 months, the nutrient intake from foods and body composition should be reassessed for improvements in diet and changes in muscle and fat mass. This three-month mark is the time when any major modifications can be made. After 12 months, bone mass and body composition can be reassessed for any changes in OSO status. A decline in OSO status would indicate that the pharmacological intervention is probably necessary.

## 8. Summary

We have shown that research into OSO is occurring in many populations globally and is even present in younger overweight/obese males and females [28]. OSO has been associated with a poor diet [20,26], a reduced functionality [11,19], frailty [12], falls [19], an unhealthier lipid profile [22] and a possible worse prognosis if present with malignancy [41]. We presented measures and cutoff values for the physical diagnosis of OSO, incorporating ectopic fat, especially visceral adipose tissue, to account for the fat redistribution into the abdominal area (occurring usually with age) and expanded the criteria to include males (Table 1) although many measures will require validation in the context of OSO. We have also discussed possible future improvements in the diagnosis of OSO, its separate components and, possibly, marrow fat as well as potential biomarkers that can be used to determine the systemic effects of OSO. Finally, we have summarized and outlined the nutritional and exercise interventions in the form of an algorithm to aid in research into the treatment and management of OSO. OSO is progressive (Figure 1), and interventions to prevent OSO are not yet defined; however, the treatment principles summarized here could be utilized in management efforts. Overall, refining the OSO criteria for measuring obesity and including options for ectopic/visceral fat will aid in future research to validate methods to diagnose OSO.

## Figures and Tables

**Table 1:** The revised physical diagnostic criteria for osteosarcopenic obesity in males and females.

Component of OSO	Males		Females	
Bone Mass	1	T-score for bone mineral density at the femoral neck, proximal femur or lumbar spine (DXA) ≤ −1.0 SD	1	T-score for bone mineral density at the femoral neck, proximal femur or lumbar spine (DXA) ≤ −1.0 SD
	2	Total Bone Mass T-score (BIA-ACC^®^) ≤ −1.0 SD	2	Total Bone Mass T-score (BIA-ACC^®^) ≤ −1.0 SD
Muscle Mass	1	Skeletal Mass Index (DXA, BIA) ≤ 5.45 kg/m^2^	1	Skeletal Mass Index (DXA, BIA) ≤ 7.26 kg/m^2^
	2	≤20th percentile of Appendicular Lean Mass (DXA, BIA)	2	≤20th percentile of Appendicular Lean Mass (DXA, BIA)
	3	S-Score (BIA-ACC^®^) ≤ −1.0 SD	3	S-Score (BIA-ACC^®^) ≤ −1.0 SD
Fat Mass	1	Total body fat (DXA, BIA) ≥ 25%	1	Total body fat (DXA, BIA) ≥ 32%
	2	Fat Mass Index ≥ 9 kg/m^2^	2	Fat Mass Index ≥ 13 kg/m^2^
Central or Visceral Fat	1	Visceral fat (CT, MRI) ≥ 130 cm^2^	1	Visceral fat (CT, MRI) ≥ 110 cm^2^
	2	Visceral/Subcutaneous fat ratio (DXA) > 1	2	Visceral/Subcutaneous fat ratio (DXA) > 1
	3	Android/Gynoid fat ratio (DXA) ≤ 1.0	3	Android/Gynoid fat ratio (DXA) ≤ 1.0
	4	Intramuscular adipose tissue (IMAT) (BIA-ACC^®^) > 2.0%	4	Intramuscular adipose tissue (IMAT) (BIA-ACC^®^) > 2.0%
	5	Waist circumference ≥ 102 cm (40 inches)	5	Waist circumference ≥ 88 cm (35 inches)
	6	Waist–hip ratio > 0.90	6	Waist–hip ratio > 0.85

OSO, Osteosarcopenic obesity; DXA, dual-energy x-ray absorptiometry; BIA, Bioelectrical Impedance Analysis; CT, Computerized Tomography; MRI, Magnetic Resonance Imaging.
